# Assessing the Detection Capacity of Microarrays as Bio/Nanosensing Platforms

**DOI:** 10.1155/2013/310461

**Published:** 2013-11-13

**Authors:** Ju Seok Lee, Joon Jin Song, Russell Deaton, Jin-Woo Kim

**Affiliations:** ^1^Bio/Nano Technology Laboratory, Institute for Nanoscience and Engineering, University of Arkansas, Fayetteville, AR 72701, USA; ^2^Department of Biological and Agricultural Engineering, University of Arkansas, Fayetteville, AR 72701, USA; ^3^Graduate Program in Cell and Molecular Biology, University of Arkansas, Fayetteville, AR 72701, USA; ^4^Department of Chemistry, Seoul National University, Seoul 151-747, Republic of Korea; ^5^Department of Statistical Science, Baylor University, Waco, TX 76798, USA; ^6^Department of Electrical and Computer Engineering, University of Memphis, Memphis, TN 38152, USA

## Abstract

Microarray is one of the most powerful detection systems with multiplexing and high throughput capability. It has significant potential as a versatile biosensing platform for environmental monitoring, pathogen detection, medical therapeutics, and drug screening to name a few. To date, however, microarray applications are still limited to preliminary screening of genome-scale transcription profiling or gene ontology analysis. Expanding the utility of microarrays as a detection tool for various biological and biomedical applications requires information about performance such as the limits of detection and quantification, which are considered as an essential information to decide the detection sensitivity of sensing devices. Here we present a calibration design that integrates detection limit theory and linear dynamic range to obtain a performance index of microarray detection platform using oligonucleotide arrays as a model system. Two different types of limits of detection and quantification are proposed by the prediction or tolerance interval for two common cyanine fluorescence dyes, Cy3 and Cy5. Besides oligonucleotide, the proposed method can be generalized to other microarray formats with various biomolecules such as complementary DNA, protein, peptide, carbohydrate, tissue, or other small biomolecules. Also, it can be easily applied to other fluorescence dyes for further dye chemistry improvement.

## 1. Introduction

Microarray detection platforms have been widely applied in various research fields of molecular biology. A microarray is one of the most powerful and successful detection platforms with its high throughput and multiplexing capability. The underlying principle of a microarray detection strategy is to estimate the number or amount of target by the interaction between immobilized probe on solid surface (e.g., glass slide or nylon membrane) and fluorescently labeled target. Since the first complementary DNA (cDNA) microarray platform was introduced [1], various microarray platforms have been explored, according to the nature of probes, such as oligonucleotide, protein, peptide, carbohydrate, tissue, or other small molecules [2–7]. However, despite the promise of microarrays as versatile biodetection platforms, most applications of microarray have been limited to genome-scale gene expression analysis, such as a preliminary screening method to investigate relative expression level of target genes or gene ontology analysis based on the accumulated amount of various genome data and their functional annotation information [8]. Hence, there is much room to expand the utility of microarray to various biological and biomedical applications [9], including environmental monitoring, pathogen detection, medical therapeutics, and drug screening, among many others, realizing the full potential of microarray.

To determine microarray's suitability as a biosensing tool for a specific application, detection sensitivity information is a critical performance index and the detection limit is one of important information, which is commonly required in the specification information of sensing devices. The detection limit is defined as “the smallest amount or concentration of particular substance that can be reliably detected in a given type of sample or medium by a specific measurement process” [10]. There are two types of analytical limits: a limit of detection (LOD) and a limit of quantification (LOQ). LOD is the minimum amount of analyte to determine its presence or absence in the sample, and LOQ is the smallest amount of analyte to be reliably estimated by the signal response value from the specific equipment or an assay procedure [11]. LOD is defined as the smallest concentration or amount that can be determined to be statistically different from a blank at 99% of confidence level [11]. Thus, the response is rarely indistinguishable from a blank at true LOD (e.g., 1%). LOD is typically determined with the data, at which the signal to noise ratio (SNR) is greater than 3 [10] and is generally accepted to be 10 times of standard deviation of the blank measure [12]. To date, there is no LOD or LOQ reported in microarray detection system. The detection sensitivity in the microarray experiment procedure has been reported as a number of transcripts per cell, when 1–5 *μ*g of target was used [13]. However, more precise LOD and LOQ are necessary to expand microarray applications as a detection tool in various research fields. As a basic index, the LOD and LOQ of microarrays are required to be defined as the smallest amount of fluorophores that can be reliably detected in a given type of printing and detection condition. 

In this study, we apply the detection limit theory and linear dynamic range to investigate the LOD and LOQ in microarray detection platform using a design for calibration with a wide spectrum of probe concentrations to estimate the limits. Also, multiple scanning was used to extend dynamic linear range [14, 15]. The detection limit theory is the experimental and analytical strategy to investigate LOD or LOQ of a specific instrument in a specific condition, using linear regression approach with statistical intervals (e.g., prediction or tolerance interval). The underlying assumption of the detection limit theory is that there should be a linear relationship between an amount of analyte (i.e., concentration) and its measured signal (i.e., response), and the smallest amount of analyte for detection and quantification can be statistically estimated by calibrating the experimental design and the relationship between known analyte amount and its response. Hubaux proposed the detection limit theory, using a spiked concentration and its prediction intervals [16]. The prediction interval is a statistical interval, which provides (1 − *α*)100% confidence of the next single observation at the true analyte concentration, whereas the confidence interval provides the reliability of an estimate with the range that includes the true value at a given confidence level. It was expected that the prediction interval could provide better estimation for the next single observation than the confidence interval. However, the nature of signals from actual experiments showed heteroskedastic trend, which means that the random variables have different variances according to their concentrations. Since the preliminary assumption of the ordinary least squares (OLS) is constant variance, it could not be applied to estimate LOD and LOQ. Later [17], Oppenheimer et al. proposed weighted least squares (WLS) with one-sided prediction interval to overcome the weakness of the OLS in practical experiment conditions. The WLS can be used to optimize the regression parameter estimation by considering proper amount of influence at each data point, that is, the more influence from the more reliable and precise data, by incorporating weights, which indicate the precision of the information of each data point. The inverse standard deviation or the inverse variance at a given concentration has been commonly used as a weight. Currie further improved the Oppenheimer's detection limit theory using linear model of standard deviation from the spiked concentration to estimate the accurate weight using standard deviation modeling [10]. Recently, Zorn et al. used prediction and tolerance intervals to assess the detection limits in sample population [11, 18]. Both statistical intervals have their own advantages. Tolerance intervals are wider and provide larger estimates of LOD and LOQ compared to equivalent prediction intervals. However, considering the estimation procedure of LOD and LOQ, which is to infer an unknown value from the known spiked samples with high degree of confidence, tolerance intervals could be better suited to the common specification information of detection tool provided by the manufacturer. Finally, to obtain precisely estimated detection limits, linear regression is a critical tool, and they should be calculated from known data in the linear range. LOD and LOQ based on the detection limit theory using linear regression with statistical intervals have been applied to various detection systems, for example, mass spectrometer for proteomic quantification [19]. However, microarray is different from other detection systems because of its expendable linear dynamic range by the adjustment of detection sensitivity. The linear dynamic range is the data range that the detector can discriminate the responses from the different concentration of a given compounds and it is linearly proportional to its concentration. To implement detection limit theory into estimation of LOD and LOQ in microarray detection platform, the dataset within linear dynamic range should be used. Here, we propose a new approach to determine LOD and LOQ in a photomultiplier-tube- (PMT-) based detection system and its sensing capability by combining the two analytical concepts, that is, linear dynamic range and detection limit theory based upon prediction and tolerance intervals using WLS. With the proposed approach, the LOD and LOQ of the most common cyanine fluorescence dyes, Cy3 and Cy5, in a microarray detection system were presented using oligonucleotide arrays as a model system. 

## 2. Materials and Methods

### 2.1. Microarray Printing, Hybridization, and Scanning

**Figure 1:**
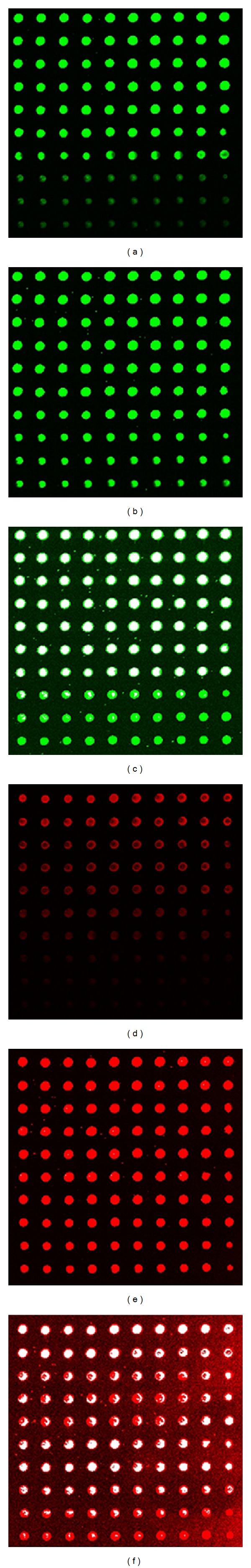
Representative microarray spot images with two common cyanine fluorescence dyes, Cy3 and Cy5, in this study. Cy3 ((a)–(c)) and Cy5 ((d)–(f)) features were scanned at various photonmultiplier tube gain (PMTG) settings: (a) PMTG 300, (b) PMTG 400, (c) PMTG 600, (d) PMTG 300, (e) PMTG 600, and (f) PMTG 800.

The 100-base oligonucleotide with 5′-amine modification (5′-TAA GTT CTT CAT ACT ATA TGT GTT CGA TGA ATT TAG TGG GTC TTC CTA AAC GTT CCT TCC ATG TTA TTG TGT TCG ATC CCA CTA GCT CCA CTT CTT CGA C-3′) was purchased from Integrated DNA Technology Inc., IA, and serial dilutions were made with printing buffer (50 mM sodium phosphate (pH 8.5)), giving a concentration range of 0 to 0.3 *μ*g/*μ*L. The 100-base oligonucleotide sequence was designed to avoid a cross-hybridization and had a minimum secondary structure according to our previously established method [20–25]. The noncrosshybridizing (NCH) sequence is thermodynamically unfavorable for hybridization to any other sequence except for their complementary strands. They were immobilized onto the CodeLink activated microarray slide (Amersham Biosciences Corp., NJ) with 10 technical replications of each concentration according to the manufacturer's instruction using MicroGridII microarray printing system and BioRobotics MicroSpot 2500 pins (Genomic Solutions, MI) at 40% relative humidity. After printing, a 40-base 3′ complementary oligonucleotide (5′-GTC GAA GAA GTG GAG CTA GTG GGA TCG AAC ACA ATA ACA T-3′), which was modified with Cy3- or Cy5-fluorophore at its 5′ end, was hybridized at the room temperature. After hybridization, the array was scanned at eight different PMTG settings in the range of 300 to 1,000 using GenePix 4000B (Axon Instruments, CA), and its signal intensity data was acquired with GenePix Pro 6.0 microarray image analysis software (Axon Instruments, CA) with 10 *μ*m of pixel size as detection sensitivity (Figure 1). The physically detected features that showed irregular spot morphology or unreasonable signal intensity were removed from the data by visual inspection. For the samples used in this study, 8 datasets were acquired from each identical sample based on the detection sensitivity. Each dataset had different dynamic ranges, that is, the range of initial printing concentration (IPC) for linear background-subtracted intensity (BSI). At the high detection sensitivity, high IPC features showed saturated BSI values. However, at the low detection sensitivity, low IPC features did not show significant BSI value for the data analysis. To obtain the data in the dynamic range, the two criteria, that is, feature saturation and SNR, were applied to the raw dataset. The features with saturation rate ≠0 and its SNR <3 were excluded from the raw dataset.

### 2.2. Data Analysis and Computations

The BSI, calculated by subtracting median background from total intensity divided by the number of pixels in feature, was used in the following statistical data analysis. Statistical analyses were performed using statistical package R (version 2.10.1) [26] with macros written to facilitate regression analyses and calculation. The LOD and LOQ were computed based on prediction and tolerance intervals using WLS to accommodate nonconstant variance of BSI, that is, significant increases of the variance of BSI as IPC increased. 

The LOD and LOQ based on the weighted prediction intervals (one-sided) are estimated by [11, 18]

(1) LOD=LC+t(1−β, n−p−2)sWb1W ×[1wLOD+1∑wi+(LOD−X¯W)2SxxW]1/2,LOQ=LQ+t(1−β, n−p−2)sWb1W ×[1wLQ+1∑wi+(LQ−X¯W)2SxxW]1/2,

where *L*_*C*_ is the critical level, *L*_*Q*_ is the determination limit, *w* is the weight at specific data, *t*_(1−*β*, *n*−*p*−2)_ is (1 − *β*)100% percentile of Student's *t*-distribution with *n* − *p* − 2 degrees of the freedom, *p* is the number of parameters used to model the weight, *b*_1*W*_ is *Sxy*_*W*_/*Sxx*_*W*_, *Sxx*_*W*_ is $\sum_{}^{}w_{i}{\left(X_{i}-{\bar{X}}_{W}\right)}^{2}$, *Sxy*_*W*_ is $\sum w_{i}(X_{i}-{\overset{-}{X}}_{w})Y_{i}$, ${\overset{-}{X}}_{w}$ is (∑*w*_*i*_ *X*_*i*_)/∑*w*_*i*_, and ${\overset{-}{Y}}_{w}$ is (∑*w*_*i*_ *Y*_*i*_)/∑*w*_*i*_. 

The LOD and LOQ based on the weighted tolerance interval are estimated by [11, 18]

(2) LOD=LC +sWb1W{t(1−β, n−2)[1∑wi+(LOD−X¯W)2SxxW]1/2     +(1wLOD)1/2N(P)(n−p−2χβn−p−22)1/2},LOQ=LQ+sWb1W{t(1−β, n−2)[1∑wi+(LQ−X¯W)2SxxW]1/2      +(1wLQ)1/2N(P)(n−p−2χβn−p−22)1/2},

where *N*(*P*) is the two-sided *P*% percentile of the standard normal distribution and ^*β*^ *χ*_*n*−*p*−2_^2^ is the (*β*)100% percentile of the *χ* ^2^ distribution with *n* − *p* − 2 degrees of the freedom. 

## 3. Results and Discussion

### 3.1. Data Filtering and Dynamic Range

There are two important data filtering parameters, pixel saturation rate (SAT) and SNR, to evaluate the feature signal quality and reliability. The first parameter is the pixel saturation within feature. Saturated pixel is caused by the physical limitation of microarray scanner in analog-to-digital image converting process and represents a status that the detected number of photons exceeds the maximum number PMT can process. At the saturated pixel, measured intensity is underestimated than its true value. The other parameter is the SNR, which has been used in various signal-detection disciplines as a criterion to determine its signal quality and reliability [27]. Pixel saturation leads to detection of the low signal data, which usually has insufficient SNR value at low PMT Gain (PMTG), and can show reasonable signal response value at high PMTG. The bottleneck of this approach is that high signal feature showed saturated pixel within its features, and this could cause the underestimation of its intrinsic signal value. Dudley et al. proposed linear regression strategy to extend the linear dynamic range of signal intensity by multiple scanning in microarray [14]. This is a useful and cost-efficient method to extract maximal information from the given experiment condition [15]. In the data analysis of this paper, raw data was processed by linear regression algorithm for extending linear dynamic range. It should be noted that physically defected features were excluded from the analysis, such as features with irregular or unexpected morphology, determined by visual inspections, that is, 115 defected features from Cy3 dataset and 49 defected features from Cy5 dataset, when total feature number of each is 560.

**Figure 2:**
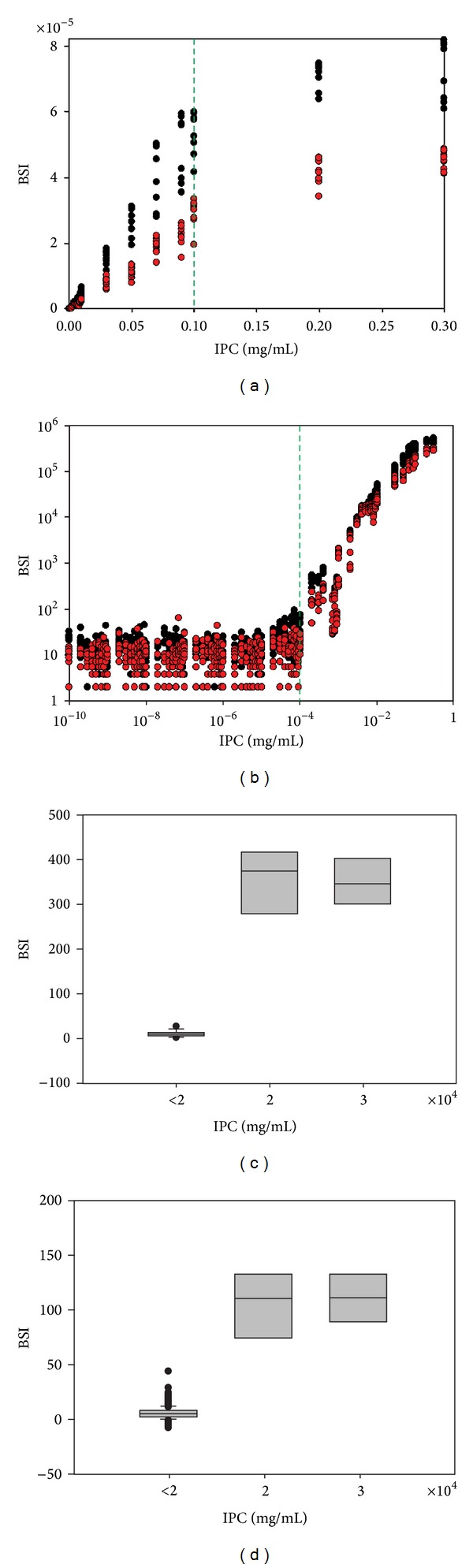
Data analyses. (a) Relationship between initial probe concentration (IPC) and background-subtracted signal intensity (BSI) in Cy3- (black circle) and Cy5-fluorophore (red circle) dataset. (b) Log-scale plot of (a) to investigate low IPC ranges. Box plots to show significant differences between BSI from low IPC (e.g., <2 × 10^−4^ of IPC) and high IPC in Cy3 (c) and Cy5 (d) dataset.

The saturated-feature-corrected BSI values were plotted to IPC (Figure 2). Overall data trend showed sigmoid type and there are two clear breakpoints, 0.1 *μ*g/*μ*L (green dotted line in Figure 2(a)) and 2 × 10^−4^ *μ*g/*μ*L (green dotted line in Figure 2(b)) of IPC. The first breakpoint was detected at 0.1 *μ*g/*μ*L of IPC using Chow test [28]. In high IPC range, it is found that BSI linearly increases up to 0.1 *μ*g/*μ*L, at which a structural break is presented. In the microarray data analysis and interpretation, signal intensity (i.e., BSI) correlated to the number of fluorophores, which is incorporated into hybridized target. Thus, the density of immobilized probe strands is very important in order to increase the signal intensity. However, too high probe density can lead to decrease of hybridization rate and cannot produce reliable signal response, which is proportional to target amount to entire target population. Strey et al. showed that double stranded DNA molecules have strong repulsions between neighbors according to decrease of the intermolecular distance [29]. To obtain the maximum signal response and extend the dynamic linear range, the immobilization of maximal number of probe in the defined area is essential. Under the proposed experiment condition, 0.1 *μ*g/*μ*L of IPC (green dotted line in Figure 2(a)) could be considered as an optimal probe density for the maximum target hybridization rate and its signal response. In low IPC range, the second breakpoint was found at 2 × 10^−4^ *μ*g/*μ*L of IPC, equivalent to 242 fluorophores/*μ*m^2^, (green dotted line in Figure 2(b)) because the delivery volume of each feature was around 700 pL with diameter of around 120 *μ*m [30]. There was clear difference between signal from 2 × 10^−4^ *μ*g/*μ*L and features smaller than that in both Cy3- and Cy5-fluorophore dataset (Figures 2(c) and 2(d)). The BSI of the low-density features, smaller than 242 fluorophores/*μ*m^2^, shows background-equivalent signals. The significance test with 95% confidence level was conducted, and the result showed that there was significant difference at 2 × 10^−4^ *μ*g/*μ*L as IPC to background-equivalent signals. In addition, the SNR values of low IPC features (lower than 2 × 10^−4^ *μ*g/*μ*L) are lower than 3, even at high PMTG. At low sensitivity setting (PMTG 300 for Cy3 and PMTG 300 and 400 for Cy5), low IPC features showed insufficient SNR for the detection. It was expected that SNR of these features could be over 3 by increasing PMTG. However, their SNR did not exceed the SNR of 3 at high PMTG. The features with its IPC between 2 × 10^−4^ *μ*g/*μ*L and 1 × 10^−3^ *μ*g/*μ*L had SNR higher than 3, but there is no linear trend of increase. These signal responses could not be included for the LOD calculation because this calibration design should show linearity between IPC and BSI and signal responses of these IPC features could not represent its IPC. This could be caused by the high variations within the repeated features, which are intrinsic problems of biological samples as well as the microarray detection system. Hence, the features, with IPC lower than 1 × 10^−3^ *μ*g/*μ*L and higher than 0.1 *μ*g/*μ*L, were removed from the further data analysis.

### 3.2. LOD and LOQ Using WLS Scheme

**Figure 3:**
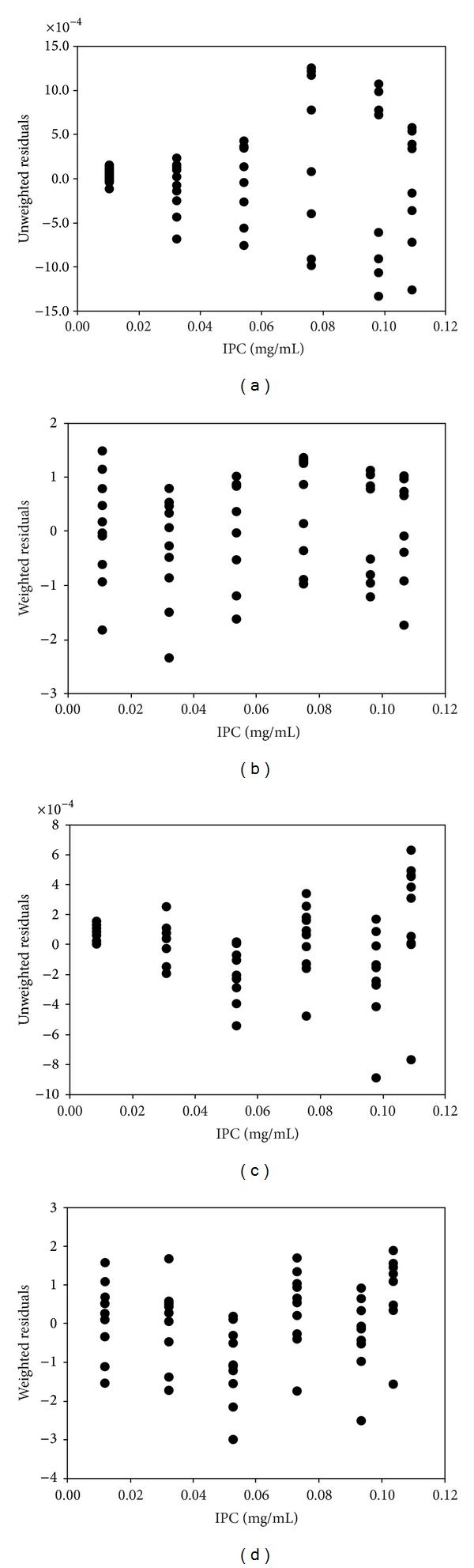
Residual plots to evaluate the heteroskadecity. Residuals obtained from the ordinary least-square method (OLS) for Cy3 (a) and for Cy5 (c), and those from the weighted least-square method (WLS) for Cy3 (b) and for Cy5 (d).

The detection limit theory requires response values in linear range and their least-square linear regression [16]. Features in the linear range were defined, as described in Section 3.1, and its linearity through lack-of-fit test was evaluated. As projected, OLS, which requires constant variance assumption, was shown to be not suitable for the microarray data analysis according to the evaluation of the homoscedasticity with the residuals from OLS (Figures 3(a) and 3(c)). The variability of BSI increased according to increase of IPC for both Cy3- and Cy5-fluorophore dataset. The funnel shape openings toward higher fitted responses (i.e., fitted BSI) in the residual plots (Figures 3(a) and 3(c)) clearly showed heteroskedastic feature. On the other hands, the residuals obtained from WLS with inverse-variance weights removed the feature, indicating that the fit accommodates the data well (Figures 3(b) and 3(d)). This justifies the use of WLS for the estimation of LOD and LOQ using the actual experimental data of microarray systems, which generally show heteroskedastic trend, that is, the random variables with different variances according to their concentrations.

In estimating the weights in WLS, it was found that both linear and quadratic models generated negative estimates of the standard deviation of a blank. Hence, to avoid the negative standard deviation, exponential model was used for the weight specification. Also limit of detection was set as 0 in the equations for LOD and LOQ to avoid their iterative solutions since it could be assumed that the limit of detection is small enough to be 0 [17].  Table 1 summarizes the LOD and LOQ estimations of Cy3 and Cy5 based on weighted prediction interval at 99% confidence (i.e., *α* = *β* = 0.01) and weighted tolerance interval at 99% confidence and 99% coverage (i.e., *α* = *β* = 0.01 and *P* = 0.99). For both fluorescence dyes, LOD and LOQ with both prediction and tolerance interval were placed within the measured dynamic range, that is, higher than the lowest end of data in the linear range. However, LOD and LOQ using tolerance intervals showed wider values than those using prediction intervals. This can be explained by the wider range of the tolerance intervals than that of the prediction intervals as reported previously [11]. Furthermore, Cy3 showed lower LOD and LOQ in both weighted prediction and tolerance intervals than Cy5. This implies that Cy3 would be more useful for both qualification and quantification than Cy5. This could be caused by the higher quantum yield of Cy3 compared to Cy5 [31]. The estimated LOD and LOQ should serve as a key index to characterize fundamental performance of the oligonucleotide microarray system using the two common fluorescence dyes. 

A number of uses of our demonstrated methodology for LOD and LOQ estimations in this study are readily envisioned. It could be directly applicable to other types of labeling dyes, such as Alexa Fluor dyes (Life Technologies, NY), which becomes available for microarray applications with high quantum yield and low photobleaching. Also, it could generalize to estimate the detection limits of different types of microarray systems, with various probes and targets including DNA, RNA, peptide, protein, carbohydrate, tissue, and other small molecules. Detection limits are considered key parameters to determine detection capability and applicability of biosensing tools. Ready assessment of ranges of detection limits would facilitate not only designs and analyses of experiments with microarray as well as other related biosensing platforms for use in specific applications but also developments of quantitative as well as qualitative models of the system. Hence, the methodology presented in this study would become an important tool to assess the detection capability of microarray and other related biosensing platforms, realizing their excellent potential as high throughput, multicolor, and multiplexing biosensing devices for various biological and biomedical applications.

## Figures and Tables

**Table 1:** Limit of detection and quantification (LOD and LOQ, resp.), which were calculated through detection limit theory with prediction and tolerance interval.

	Weighted prediction intervals^a^		Weighted tolerance intervals^b^	
	LOD	LOQ	LOD	LOQ
	Number of fluorophores/*μ*m^2^			
Cy3	13,366	36,606	21,046	42,666
Cy5	16,487	39,680	26,047	46,440

^a^99% confidence (i.e., *α* = *β* = 0.01).

^b^99% confidence and 99% coverage (i.e., *α* = *β* = 0.01 and *P* = 0.99).
